# Feasibility of Indirect Secondary Distribution of HIV Self-test Kits via WeChat Among Men Who Have Sex With Men: National Cross-sectional Study in China

**DOI:** 10.2196/28508

**Published:** 2021-10-26

**Authors:** Shangcao Li, Jing Zhang, Xiang Mao, Tianyi Lu, Yangyang Gao, Wenran Zhang, Hongyi Wang, Zhenxing Chu, Qinghai Hu, Yongjun Jiang, Wenqing Geng, Hong Shang, Junjie Xu

**Affiliations:** 1 NHC Key Laboratory of AIDS Immunology National Clinical Research Center for Laboratory Medicine The First Affiliated Hospital of China Medical University Shenyang，Liaoning Province China; 2 Key Laboratory of AIDS Immunology Chinese Academy of Medical Sciences Shenyang China; 3 Key Laboratory of AIDS Immunology of Liaoning Province Shenyang China

**Keywords:** secondary distribution, HIV, men who have sex with men, WeChat, HIV self-testing

## Abstract

**Background:**

HIV self-testing (HIVST) kits are common in key sexually active populations. Direct secondary distribution of HIVST kits (DSDHK) is effective in improving the uptake of HIVST. However, there are concerns about the various limitations of DSDHK, including limited geographic reach, payment problems, and need for face-to-face interactions.

**Objective:**

In this study, we aim to evaluate the feasibility and characteristics of indirect secondary distribution of HIVST kits (ISDHK) via WeChat (distributing HIVST application links and follow-up HIVST kits to partners) among men who have sex with men (MSM).

**Methods:**

From October 2017 to September 2019, an HIVST recruitment advertisement was disseminated on the WeChat social media platform to invite MSM to apply for the HIVST kits (referred to as *index participants* [IPs]). All MSM participants were encouraged to distribute the HIVST application link to their friends and sexual partners (referred to as *alters*) through their social networks. All the alters were further encouraged to continue distributing the HIVST application link. All participants paid a deposit (US $7), which was refundable upon completion of the questionnaire, and uploaded the test results via a web-based survey system.

**Results:**

A total of 2263 MSM met the criteria and successfully applied for HIVST. Of these, 1816 participants returned their HIVST results, including 1422 (88.3%) IPs and 394 (21.7%) alters. More alters had condomless anal intercourse, a higher proportion of them had never previously tested for HIV, and they showed a greater willingness to distribute HIVST kits to their sexual partners (*P*=.002) than the IPs. After controlling for age, education, and income, the alters had a greater proportion of MSM who had never tested for HIV before (adjusted odds ratio [aOR] 1.29, 95% CI 1.00-1.68), were more willing to distribute the HIVST application link (aOR 1.71, 95% CI 1.21-2.40), had a lower number of sexual partners (aOR 0.71, 95% CI 0.57-0.90), and were less likely to search for sexual partners on the web (aOR 0.78, 95% CI 0.60-1.02) than IPs. In comparison, the rates of reactive HIVST results, conducting HIV confirmatory tests, HIV seropositivity, and initiation of HIV antiretroviral therapy were similar for IPs and alters.

**Conclusions:**

The ISDHK model of distributing HIVST application links among the MSM population via social media is feasible. The ISDHK model should be used to supplement the DSDHK model to enable a greater proportion of the MSM population to know their HIV infection status.

## Introduction

### Background

HIV testing is the first step in HIV treatment and care and is a crucial part of HIV prevention [1]. However, as of 2019, approximately 20% of people living with HIV globally still do not know their serological status [2], and this figure is approximately 25% in China. Men who have sex with men (MSM) continue to be disproportionately impacted by the HIV pandemic [3]. The proportion of MSM among the annual newly reported HIV infections in China increased from 16% in 2011 to 23.4% in 2018 [4,5]. A national survey in 2016 showed that only 47% of Chinese MSM had ever tested for HIV, and only 38% had tested for HIV in the last 12 months [6]. There are barriers to traditional facility-based HIV testing for MSM, including concerns about discrimination, privacy, time, and transportation [7-9]. Hence, increasing people’s awareness of HIV status through innovative methods has become the focus of HIV prevention campaigns globally.

HIV self-testing (HIVST) is a new strategy recommended by the World Health Organization to improve the uptake of HIV testing among key populations [10]. Evidence shows that HIVST has the potential to overcome facility-based barriers to HIV testing, improve testing conditions in areas where HIV testing opportunities are insufficient, and increase accessibility to testing [11].

Direct secondary distribution of HIVST kits (DSDHK) is a social network–based approach, giving *index participants* (IPs) multiple HIVST kits for direct distribution to their sexual partners or others in their social network (referred to as *alters*) [12]. Several studies have demonstrated greater acceptability and effectiveness of DSDHK, in which IPs share HIVST kits with alters. In South Africa and Uganda, 2 cohort studies showed that DSDHK is acceptable and feasible for MSM, increasing the frequency of HIV testing among MSM and improving early detection of HIV [13,14]. In Kenya, Uganda, and South Africa, 4 randomized controlled trials showed that DSDHK promoted HIV testing by helping MSM and female sex workers to adopt safer sex practices with their sexual partners through secondary distribution of self-test kits by HIV-negative female sex workers and women receiving antenatal and postpartum care [12,15-17]. A recent study conducted in China, which used social media to recruit IPs, showed that DSDHK increased the level of diagnosis of HIV-positive cases and the coverage of HIV testing among MSM [18]. However, the abovementioned DSDHK study recruited only participants living around the study sites, and only a limited number of alters were able to obtain HIVST kits from IPs. The sharing of HIVST kits requires the IPs to live in the same city as alters or for it to be shared by mail, which will arouse various concerns, including concerns regarding geographic location for distributing HIVST kits, payment for mailing HIVST kits, or face-to-face interaction [19]. This is an instance of a lack of innovative social determinants of health theory, which highlights the limitations of DSDHK practice. Moreover, no studies that we are aware of have explored the feasibility and effectiveness of an indirect secondary distribution of HIVST kits (ISDHK) model of HIVST recruitment and self-test kit distribution.

WeChat is the most popular mobile social media platform in use in China today, with more than 1 billion registered user accounts [20]. We hypothesize that an ISDHK model could boost the distribution of HIVST among MSM who were willing to promote HIV testing to their sexual partners or friends by sharing HIVST application links (websites or QR codes for web-based applications, mailing, and uploading of test results).

### Objectives

This study aims to evaluate the feasibility of social media–based ISDHK among MSM and explore differences in sexual behaviors, HIV testing behaviors, willingness to distribute HIV testing further, and linkage-to-HIV care characteristics between IPs and alters.

## Methods

### Study Setting and Participants

From October 2017 to September 2019, we performed a WeChat-based cross-sectional study of ISDHK among MSM in mainland China (Hong Kong, Macao, and Taiwan were not included). Participants were eligible if they were male, aged ≥16 years, had reported having engaged in anal sex with another man on at least one occasion, willing to provide their sexual behavior information, and willing to apply for an HIVST kit for HIVST, provide the results, and sign the digital informed consent form.

The ISDHK research was carried out in Shenyang, Liaoning Province, China (First Affiliated Hospital of China Medical University).

### IP Recruitment

The recruitment advertisements for this HIVST study were disseminated by 6 key opinion leaders of MSM through 6 WeChat public accounts, and detailed information about the participants’ recruitment was published [21]. The primary subscribers to the 6 WeChat public accounts were from the MSM population. The advertisements included an introduction to and information about informed consent and links to apply for HIVST. Those MSM interested in the HIVST project who successfully completed the application questionnaire were provided with HIVST kits (the IPs) and encouraged to share the HIVST kit application links with their peer MSM friends and sexual partners.

### Indirect Secondary Distribution of HIVST Kits

IPs were recruited through the 6 WeChat public accounts and encouraged to share the HIVST service links on social media (such as Blued, WeChat Moments, etc) or with their sexual partners and friends (the *alters*). The alters obtained HIVST via the HIVST application link on various social media platforms shared by IPs. The alters were also encouraged to further share the HIVST application link on the social networks that they used.

### Provision of HIVST Kits

**Figure 1:**
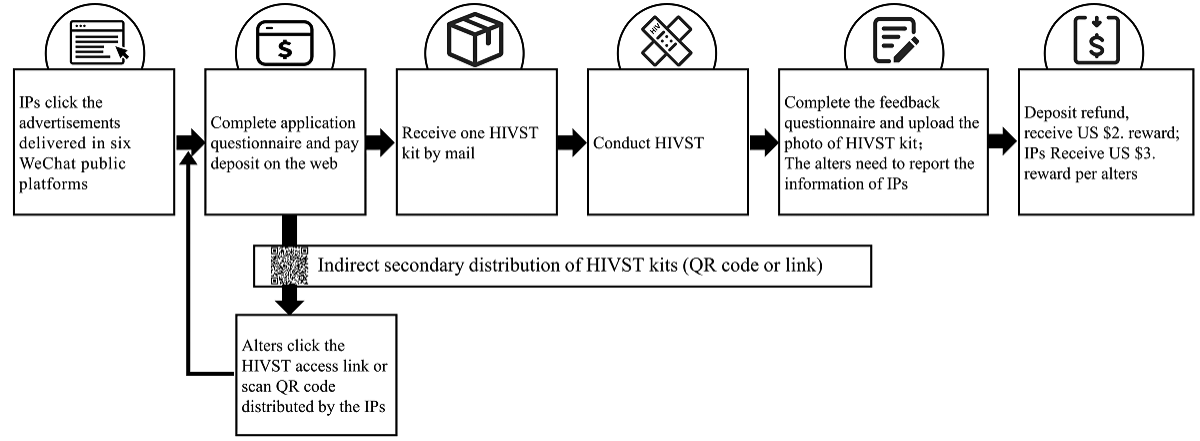
WeChat-based model of indirect secondary distribution of HIV self-testing kits. HIVST: HIV self-testing; IP: index participant.

Following the completion of their HIVST applications, information about participants’ sociodemographic and sexual behaviors was collected by means of *Golden Data*, a web-based survey software (Xi’an Data Rujin Information Technology Co, Ltd). The study investigators reviewed the eligibility of the HIVST applications from all applicants. Then, to ensure the uploading of a proportion of HIVST test results, the enrolled eligible MSM participants were asked to pay a deposit of US $7, which would be refunded following the uploading of the test results and conducting of the HIV posttesting counseling. Fingerstick whole blood HIVST kits, and supporting tools (a disposable retractable blood needle, capillary tube, Wondlfo solution, alcohol tables, disposable fingertip blood collection needle, woundplast, and condoms) were sent to eligible participants by express mail. A reward of US $2 was paid to participants who uploaded their test results to compensate them for their time spent participating in the survey and to increase the feedback rate of HIVST results. We also asked the alters to fill in the IP information (phone number, WeChat name, or name) to understand the distribution network and pay fees as an incentive. IPs received an additional reward of US $3 if they helped recruit an alter to attend the HIVST project and upload their HIVST results (Figure 1).

### Postcounseling, Linkage-to-Care, and Follow-up

If a participant had a *positive* or *indeterminate* HIVST test result, they were immediately contacted by trained researchers for an HIV posttest consultation via WeChat or over the phone to help with the interpretation of the test results and referral to services for clinical confirmatory testing and antiviral treatment. If the participant was located in Shenyang or surrounding cities, they were invited to the Voluntary Counseling and Test of the First Hospital of China Medical University to provide 10 mL of venous blood for HIV confirmation, diagnosis, and treatment. The local Chinese Center for Disease Control information about further testing was also provided to them.

### Measurements

After conducting the HIVST, participants were required to provide feedback on their HIVST results by uploading the HIVST result pictures and completing a computer-assisted self-administered questionnaire. The web-based questionnaire system is equipped with logic verification, which automatically checks the survey object before it is submitted, avoiding missing numbers or logic errors. The questionnaire included questions about the following: (1) sociodemographic information, including age, occupation, educational level, and monthly income; (2) sexual behavior, including ways of finding sexual partners, sexual behavioral roles with other males, numbers of sexual partners, questions about condomless anal intercourse (CAI), and questions about chemsex (a term used by MSM to describe sex that occurs under the influence of drugs such as rush [poppers or alkyl nitrites], MDMA [3,4-methylenedioxymethamphetamine; ecstasy], ice, amphetamines, tramadol, or ketamine) in the last 6 months; (3) HIV testing, including history of HIV testing, types of HIV testing, and frequency of HIV testing; (4) photo of the HIVST result uploaded to the system; and (5) the source of their test paper for this test. If the participants reported, “Apply through the link shared by your friends” or “Your friends help you apply,” they were identified as alters and were asked to report the IPs’ names and phone numbers. IPs and alters were asked to complete the same questionnaire.

### Sample Size

We calculated the sample size of MSM participants using a simple random sampling method to test 2 independent proportions. According to the preliminary study, the ratio of alters to IPs is 0.3, the estimated share of IPs who had never tested for HIV was 20% and the estimated share of alters who had never tested for HIV was 30%. We used the parameters of 80% power at a 2-sided significance level of .05 and calculated that the smallest sample size was 600 IPs and 200 alters. We used PASS (Power Analysis and Sample Size) software, version 11 (NCSS) to calculate the sample size.

### Statistical Analysis

Quantitative variables are classified; for example, age is divided into <24 years and ≥24 years, and the number of sexual partners is divided into <2 and >2. The distribution of demography, sexual behavior, and HIV testing–related characteristics of IPs and alters was compared using chi-square analysis or Fisher exact test. A multivariable logistic regression model was used to analyze the correlation between alters and IPs. Age, educational level, and monthly income were adjusted in the multivariable logistic model. Data analysis was performed using SPSS 25.0 software (IBM Corporation). Two-way *P* values that were <.05 were considered to be statistically significant. See the STROBE (Strengthening the Reporting of Observational Studies in Epidemiology) checklist for this cross-sectional study in Multimedia Appendix 1.

### Ethical Permission

This study was approved by the Institutional Review Board of the First Affiliated Hospital of China Medical University (Project No. 2018-174-2). A signed digital informed consent form was obtained from all the participants before collecting any study information or specimens. Participants joined the study voluntarily and were free to withdraw from the study at any time. When collecting data through the *Golden Data* web-based survey platform, only the researcher was provided with an account and password to ensure the participants’ data security and privacy. The data were downloaded directly and saved on a local computer. Identifiable variables (such as nicknames, phone numbers, and addresses) were encrypted to protect the privacy of the participants. HIV test results were disclosed only to the person concerned and not to anyone else.

## Results

### Sociodemographic Characteristics of the Participants

**Figure 2:**
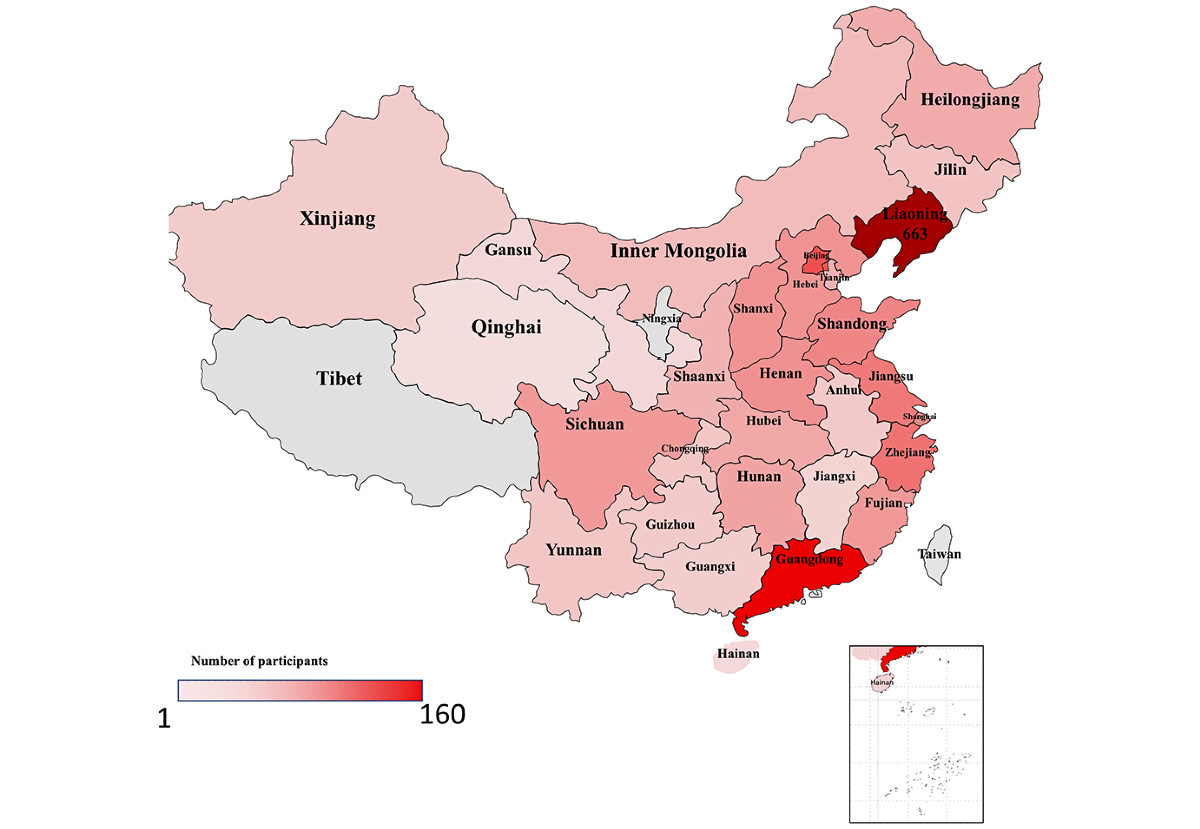
Distribution of participants in 29 provinces of China who fed back their HIV self-testing.

**Figure 3:**
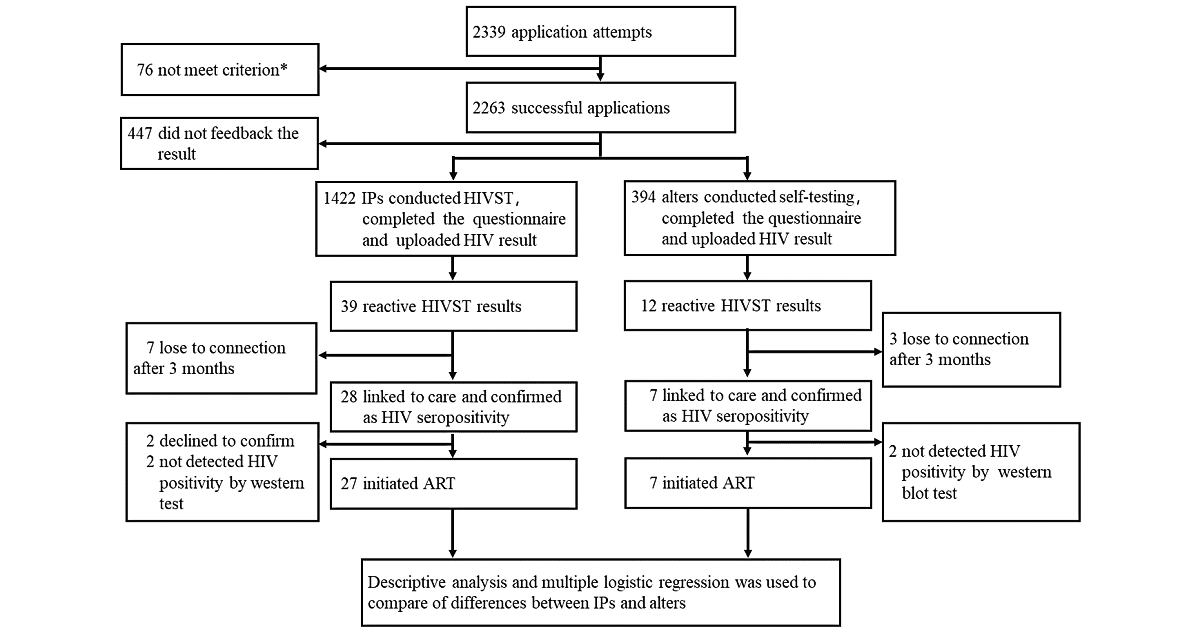
Flowchart of results and linkage to care of HIV self-testing secondary distribution indirectly among men who have sex with men in 29 provinces in China, 2017-2019. “*” indicates participants were eligible and paid the deposit; ART: antiretroviral therapy; HIVST: HIV self-testing; IP: index participant.

In total, 2263 MSM participants attended the HIVST project and applied for the HIVST kits. Of these, 80.25% (1816/2263) were unique participants from 29 provinces and 229 cities in mainland China (Figure 2) who completed the feedback questionnaire and uploaded their HIVST results. A total of 1816 eligible MSM were included in this cross-sectional study, of which 78.3% (1422/1816) were categorized as IPs and 21.69% (394/1816), as alters. Alters were recruited by IPs through ISDHK (Figure 3).

Table 1 summarizes the sociodemographic characteristics, sexual behaviors, and HIV testing behaviors of alters and IPs. Alters were aged ≥24 years (251/394, 64.3%), had college-level or higher-level education (320/394, 81.2%), sought sexual partners through offline means (303/394, 76.9%), had more than 2 sexual partners (220/394, 55.8%), had been tested for HIV before (283/394, 71.8%), were willing to share HIVST results with sexual partners (349/394, 88.6%), and applied regularly for HIVST (353/394, 89.6%; Table 1).

**Table 1:** Demographic characteristics, sexual behaviors, and HIV prevalence of index participants and alters (N=1816).

Characteristic			Alters (n=394), n (%)		Index participants (n=1422), n (%)
Age above 24 years			251 (63.7)		918 (64.6)
**Monthly income (US $)**					
	<300	125 (32.6)		453 (31.9)	
	300-900	168 (43.8)		550 (38.7)	
	900	91 (23.7)		361 (25.4)	
Having a university degree or above			320 (81.2)		1239 (87.1)
Student			129 (32.7)		478 (33.6)
Homosexual			265 (67.3)		992 (69.8)
**Sexual role**					
	Insertive	125 (31.7)		423 (29.7)	
	No preference	111 (28.2)		389 (27.4)	
	Acceptive	115 (29.2)		443 (31.2)	
	Oral	43 (10.9)		167 (11.7)	
**Sexual behavior in the last six months**					
	Sought sexual partners on the web	303 (76.9)		1151 (80.9)	
	Had >2 sexual partners	220 (55.8)		889 (62.5)	
	Engaged in commercial anal intercourse	8 (2)		37 (2.6)	
	Engaged in chemsex^a^	145 (36.8)		50 (35.5)	
	Engaged in group sex with males	32 (8.1)		123 (8.6)	
	Engaged in CAI^b^ with males	184 (46.7)		580 (40.8)	
	Experienced anal bleeding	134 (34)		498 (35)	
	Experienced symptoms of an STI^c^	33 (8.4)		170 (12)	
**HIV testing frequency**					
	Never tested for HIV before	111 (28.2)		329 (23.1)	
	Every 3 months or more often	53 (18.7)		216 (20)	
	Every 6 months	77 (27.2)		338 (31)	
	Every 12 months	67 (23.7)		219 (20)	
	Every ≥12 months	18 (6.4)		71 (6.5)	
	No regular frequency	68 (24)		249 (22.8)	
Willingness to share HIVST^d^ with sexual partners			349 (88.6)		1167 (82.1)
Apply for HIVST regularly			353 (89.6)		1306 (91.8)
Positive HIVST result			12 (3)		39 (2.7)
**HIV confirmatory test**					
	Positive	7 (58.3)		28 (71.8)	
	Negative or unknown	5 (41.7)		6 (28.2)	

^a^Used rush (poppers or alkyl nitrites), MDMA (3,4-methylenedioxymethamphetamine; ecstasy), ice, amphetamines, tramadol, or ketamine in the past six months.

^b^CAI: condomless anal intercourse.

^c^STI: sexually transmitted infection.

^d^HIVST: HIV self-testing.

### Differences Between IPs and Alters

Chi-square analysis showed that alters had a higher likelihood of having engaged in CAI (184/394, 46.6% vs 580/1422, 40.78%; *P*=.04), a higher proportion of never having previously tested for HIV (111/394, 28.2% vs 329/1422, 23.14%; *P*=.04), and greater willingness to recommend or promote HIVST to sexual partners (349/394, 88.6% vs 1167/1422, 82.07%; *P*=.002) than IPs (Multimedia Appendix 2).

Multivariable logistic regression analysis showed that alters had fewer sexual partners (adjusted odds ratio [aOR] 0.71, 95% CI 0.57-0.90), a lower proportion with symptoms of sexually transmitted infections (aOR 0.67, 95% CI 0.45-1.00), a greater willingness to distribute the HIVST application link to partners and friends (aOR 1.71, 95% CI 1.21-2.40), a marginally higher proportion of never having previously tested for HIV (aOR 1.29, 95% CI 1.00-1.68), and a marginally lower proportion of MSM seeking sexual partners through offline means (aOR 0.78, 95% CI 0.60-1.02; Multimedia Appendix 2).

### HIVST Results and Linkage-to-Care

Overall, out of the 1816, 51 (2.81%) study participants had a reactive HIVST result. Of these, 39 were IPs and 12 were alters. The reactive HIV rates among the 2 groups showed no significant differences (39/1422, 2.74% vs 12/394, 3%; *P*=.75). Among those who recorded a reactive HIVST result, the percentage of participants who sought HIV care and were followed up was similar between IPs and alters (32/39, 82% vs 9/12, 75%; *P*=.70). The percentages of participants who had an HIV-positive confirmatory test (28/32, 93% vs 7/9, 77%; *P*=.99), and who initiated HIV ART (antiretroviral therapy; 27/28, 96% vs 7/7, 100%; *P*=.99) were also similar between IPs and alters (Table 2).

**Table 2:** The proportions of index participants and alters who accessed different HIV testing services.

HIV care cascade	Index participants, n (%)	Alters, n (%)	*P* value^a^
Reactive HIVST^b^	39 (2.7)	12 (3)	.75
Follow-up	32 (82)	9 (75)	.70
Confirmatory testing	28 (88)	7 (78)	.99
Initiate ART^c^	27 (96)	7 (100)	.99

^a^Fisher exact test.

^b^HIVST: HIV self-testing.

^c^ART: antiretroviral therapy.

## Discussion

### Principal Findings

To the best of our knowledge, this study is the first to clarify that the indirect secondary distribution of HIVST application links through the WeChat social media app is feasible and effective for expanding the coverage of HIV testing among the MSM population in China. This new ISDHK model can reach out to those MSM who have a limited HIV testing history and a higher likelihood of having engaged in CAI. The ISDHK model can expand the coverage of HIV testing among the social networks of MSM. The rates of HIVST-positive reactive results, HIV seropositivity, and the linkage-to-care ratio of MSM recruited by ISDHK were similar to those directly recruited from the MSM population. This finding indicates that ISDHK could fill in the gaps in our knowledge of DSDHK theory and practice.

Our study found that the ISDHK model increased the coverage of HIVST distribution to MSM partners and friends on the internet among MSM participants. However, DSDHK can continue to expand the continued distribution of HIVST among key populations through the social networks of participants and increase the coverage of HIV testing. However, the direct method faces the limitation of limited distribution. Most HIVST kits can only be distributed to friends and sexual partners in person. The influence of the DSDHK is relatively limited, and it is difficult to influence some key groups. Instead of providing the HIVST kit directly to MSM, we provided a social media–based HIVST service link, which could be easily forwarded via the social networks of MSM. We encouraged all recruited MSM to share the HIVST service link to help their partners receive HIVST kits. Through voluntary application and mailing, HIVST kits can reach beyond the recruited participants and expand the testing coverage to wider social networks [22]. In addition, the indirect model has the advantage of helping the participants avoid face-to-face interactions and associated challenging encounters, such as partner violence faced by the participants of DSDHK [23]. During the global COVID-19 pandemic, both MSM and medical workers need to maintain physical distancing, and facility-based HIV testing services are restricted, making it more difficult for MSM to obtain good facility-based HIV testing services [24]. In addition, the ISDHK model can access a greater number of MSM from 29 different provinces and municipalities across China than facility-based HIV tests; whereas facility-based HIV tests provide more accurate testing and better consulting services, 1 facility-based testing center is usually only suitable for the region around the city in which it is based [17]. Combining direct and indirect secondary distribution models by giving MSM multiple HIVST kits and application links may influence more MSM to get tested for HIV. Hence, it is necessary to integrate the ISDHK model into the HIV testing service to improve the coverage of HIV testing.

To the best of our knowledge, this study is the first to find that a higher proportion of MSM who received ISDHK had never previously been tested for HIV and preferred to find sexual partners via offline means. MSM who received ISDHK were more likely to have never previously been tested for HIV than those who received DSDHK (111/394, 28.2% vs 329/1422, 23.14%). The results of this study are consistent with those of Wu et al [18] obtained in Guangdong, China, through social networks for DSDHK (IPs: 21% vs alters: 40%). The ISDHK model may help MSM who have never been tested for HIV, thus reducing HIV transmission. A meta-analysis and previous studies have shown that MSM who find sexual partners via offline means are generally older and have lower HIV test rates, higher CAI incidence, and higher HIV prevalence [25-27]. Hence, providing HIV testing services and interventions to MSM who seek male sexual partners offline is very important for the prevention and control of HIV transmission in the overall service of public health. The results of this study indicate that ISDHK plays an essential role in expanding the coverage of HIV testing and in interventions for high-risk sexual practices (for HIV) among MSM. It is crucial to increase the coverage and frequency of HIV testing for MSM seeking male sexual partners offline and to further reduce high-risk sexual behavior.

This study also describes the HIV linkage-to-care characteristics of the MSM received ISDHK. There is no statistically significant difference in the HIV-linkage-to-care rates between MSM who received secondary distribution and those directly recruited via WeChat (27/28, 96% vs 7/7, 100%). These rates are higher than the HIV-positive referral rates of a social network–based DSDHK in Guangdong (12/15, 80%) [18] and a randomized controlled trial study in Uganda, where a women’s facility-based study delivered HIVST kits to their male partners (6/26, 23%) [28]. A vast majority of MSM who were confirmed to be positive began ART within 3 months. This may have been for the following reasons: following a reactive HIVST result, using the working platform of this project, the staff were able to provide immediate web-based HIV testing consultation and linkage-to-care services through WeChat and participants suspected of being HIV-infected were recommended to facility-based tests in order to provide them with free HIV re-examination, confirmation, and ART. This study showed that the ISDHK model operated via WeChat could increase the rate of HIV testing and promote the completion of the first third of the 90% global target and the second and third AIDS prevention and control targets. Although this study and previous similar studies in China have shown that ISDHK has obvious advantages in improving the coverage of HIV testing and can compensate for the limitations of traditional facilities-based HIV testing, the current Chinese HIVST guidelines have no relevant technical specifications or guidance on ISDHK [29]. The results of this study provide the data that support the updating of the Chinese HIVST guidelines, with the aim of increasing the geographic and societal distribution of HIVST.

### Strengths and Limitations

There are 3 strengths of this study. This is the first national-wide study carried out on the WeChat social media platform using ISDHK among MSM in China. Second, it is the first study to report the behavioral characteristics of alters among MSM. Third, this study is the first to encourage participants to distribute HIVST among their social media.

There are 3 limitations of this study. First, approximately one fifth of the survey respondents did not return their HIV results after receiving the self-test kit. Therefore, there may be reporting bias, and the results of this study did not reflect the characteristics of those who did not feedback their HIVST results and this may lead to a lower HIV-positive rate. Further research is needed to improve the feedback rate of the results from those participating in the HIVST. Second, only 76.6% (302/394) of participants reported 246 IPs’ information; therefore, information about the complete secondary distribution network is unknown. Third, each participant could obtain only one HIVST kit for each application and pay a Chinese ¥ 50 deposit (approximately US $7), thus incentivizing the participant to feed back their test results. However, poorer participants may be discouraged or even prevented from applying for HIVST. Therefore, there may be selection bias, and the requirement for paying this deposit may have affected the representativeness of the results.

### Conclusions

The indirect secondary distribution model of distributing HIVST application links via the WeChat social media platform is feasible for the MSM population. It can increase the coverage of HIV testing among MSM, especially among those who have a history of not being tested for HIV. It is necessary to integrate DSDHK with ISDHK to help increase the coverage of HIVST among MSM and other key populations.

## Appendix

Multimedia Appendix 1 The STROBE (Strengthening the Reporting of Observational Studies in Epidemiology) checklist for this cross-sectional study.

Multimedia Appendix 2 Comparison between index participants and alters (N=1816).

## Abbreviations

aOR: adjusted odds ratio
ART: antiretroviral therapy
CAI: condomless anal intercourse
DSDHK: direct secondary distribution of HIV self-testing kits
HIVST: HIV self-testing
IP: index participant
ISDHK: indirect secondary distribution of HIV self-testing kits
MSM: men who have sex with men
PASS: Power Analysis and Sample Size
STROBE: Strengthening the Reporting of Observational Studies in Epidemiology
