# Supplementary material for: Feasibility of Indirect Secondary Distribution of HIV Self-test Kits via WeChat Among Men Who Have Sex With Men: National Cross-sectional Study in China
Source: J Med Internet Res. 2021 Oct 26;23(10):e28508. doi: 10.2196/28508 (PMC8579221; doi:10.2196/28508)
Supplement: Multimedia Appendix 2 [file jmir_v23i10e28508_app2.docx]

**Multimedia Appendix 2.**

**Table 3.** Comparison between index participants and alters (N=1816).

| Characteristic | | | Alters, n (%) | cOR^a^ (95% CI) | *P* value | aOR^b^ (95% CI) | Adjusted *P* value^c^ |  |
| --- | --- | --- | --- | --- | --- | --- | --- | --- |
| **Demographic information** | | | | | | | | |
|  | **Age (years)** | | | | | | | |
|  |  | <24 | 143 (22.1) | 1.04 (0.82-1.31) | .76 | —^d^ | — | |
|  |  | ≥24 | 251 (21.5) | Reference | — | — | — | |
|  | **Monthly income (US $)** | | | | | | | |
|  |  | <300 | 125 (21.6) | 0.91 (0.67-1.24) | .56 | — | — | |
|  |  | 300–900 | 168 (23.4) | 0.83 (0.62-1.10) | .19 | — | — | |
|  |  | 900 | 91 (20.1) | Reference | — | — | — | |
|  | **Education** | | | | | | | |
|  |  | Senior high school or below | 74 (28.8) | 1.57 (1.16-2.11) | .003 | — | — | |
|  |  | College or above | 320 (20.5) | Reference | — | — | — | |
|  | **Occupation** | | | | | | | |
|  |  | Student | 129 (21.3) | 0.96 (0.76-1.22) | .75 | 1.04 (0.71-1.53) | .84 | |
|  |  | Other | 265 (21.9) | Reference | — | Reference | — | |
|  | **Sexual orientation** | | | | | | | |
|  |  | Homosexual | 265 (21.1) | 0.89 (0.7-1.13) | .34 | 0.87 (0.68-1.11) | .28 | |
|  |  | Other | 129 (23.1) | — | — | Reference | — | |
|  | **Sexual role** | | | | | | | |
|  |  | Insertive | 125 (22.8) | 1.04 (0.77-1.38) | .81 | 1.05 (0.78-1.41) | .75 | |
|  |  | No preference | 111 (22.2) | 1.14 (0.86-1.52) | .38 | 1.1 (0.83-1.48) | .51 | |
|  |  | Acceptive | 115 (20.6) | 1.15 (0.78-1.7) | .49 | 1.11 (0.75-1.65) | .61 | |
|  |  | Oral | 43 (20.5) | Reference | — | Reference | — | |
| **Sexual behavior in the previous six months** | | | | | | | | |
|  | **Means for seeking sexual partners** | | | | | | | |
|  |  | On the web | 303 (20.8) | 0.78 (0.6-1.03) | .08 | 0.78 (0.59-1.03) | .08 | |
|  |  | Offline | 91 (25.1) | Reference | — | Reference | — | |
|  | **Number of sexual partners** | | | | | | | |
|  |  | <2 | 174 (24.6) | 1.32 (1.05-1.65) | .02 | 0.71 (0.57-0.9) | .004 | |
|  |  | ≥2 | 220 (19.8) | Reference | — | Reference | — | |
|  | **Engage in commercial anal intercourse** | | | | | | | |
|  |  | Yes | 8 (17.8) | 0.78 (0.36-1.68) | .52 | 0.78 (0.36-1.69) | .53 | |
|  |  | No | 386 (21.8) | Reference | — | Reference | — | |
|  | **Engage in chemsex**^e^ | | | | | | | |
|  |  | Yes | 145 (22.3) | 1.06 (0.84-1.33) | .64 | 1.02 (0.81-1.3) | .84 | |
|  |  | No | 249 (21.4) | Reference | — | Reference | — | |
|  | **Engage in group sex with males** | | | | | | | |
|  |  | Yes | 32 (20.6) | 0.93 (0.62-1.4) | .74 | 0.89 (0.59-1.35) | .59 | |
|  |  | No | 362 (21.8) | Reference | — | Reference | — | |
|  | **Engage in CAI**^f^ **with males** | | | | | | | |
|  |  | Yes | 184 (24.1) | 1.27 (1.02-1.59) | .04 | 1.22 (0.97-1.53) | .10 | |
|  |  | No | 210 (20) | Reference | — | Reference | — | |
|  | **Have experienced anal bleeding** | | | | | | | |
|  |  | Yes | 134 (21.2) | 0.96 (0.76-1.21) | .71 | 0.93 (0.73-1.19) | .58 | |
|  |  | No | 260 (22) | Reference | — | Reference | — | |
|  | **Ever had symptoms of an STI**^g^ | | | | | | | |
|  |  | Yes | 33 (16.3) | 0.67 (0.46-1) | .046 | 0.67 (0.45-1) | .049 | |
|  |  | No | 361 (22.4) | Reference | — | Reference | — | |
| **HIV testing** | | | | | | | | |
|  | **Never tested for HIV before** | | | | | | | |
|  |  | Yes | 111 (25.2) | 1.3 (1.01-1.68) | .04 | 1.29 (1-1.68) | .05 | |
|  |  | No | 283 (20.6) | Reference | — | Reference | — | |
|  | **HIV testing frequency** | | | | | | | |
|  |  | Every 3 months or more often | 53 (19.7) | 1.2 (0.83-1.73) | .33 | 1.24 (0.85-1.79) | .26 | |
|  |  | Every 6 months | 77 (18.6) | 0.89 (0.61-1.31) | .56 | 0.87 (0.59-1.28) | .47 | |
|  |  | Every 12 months | 67 (23.4) | 1.11 (0.74-1.66) | .60 | 1.11 (0.74-1.67) | .61 | |
|  |  | Every ≥12 months | 18 (20.2) | 1.08 (0.6-1.93) | .80 | 0.98 (0.54-1.77) | .94 | |
|  |  | No regular frequency | 68 (21.5) | Reference | — | Reference | — | |
|  | **Willing to share HIVST**^h^ **with sexual partners** | | | | | | | |
|  |  | Yes | 349 (23) | 1.69 (1.21-2.38) | .002 | 1.7 (1.21-2.4) | .002 | |
|  |  | No | 45 (15) | Reference | — | Reference | — | |
|  | **Apply for HIVST regularly** | | | | | | | |
|  |  | Yes | 353 (21.3) | 0.76 (0.53-1.11) | .16 | 0.74 (0.51-1.09) | .13 | |
|  |  | No | 41 (26.1) | Reference | — | Reference | — | |
|  | **HIVST result** | | | | | | | |
|  |  | Positive | 12 (23.5) | 1.11 (0.58-2.15) | .75 | 0.91 (0.47-1.77) | .79 | |
|  |  | Negative | 382 (21.6) | Reference | — | Reference | — | |
|  | **HIV confirmatory test** | | | | | | | |
|  |  | Positive | 7 (20) | 0.62 (0.16-2.36) | .39 | 0.6 (0.15-2.36) | .47 | |
|  |  | Negative or unknown | 5 (45.5) | Reference | — | Reference | — | |

^a^cOR: crude odds ratio.

^b^aOR: adjusted odds ratio.

^c^Logistic regression adjusted for age (years), income (US $), and education.

^d^Adjusted factor or reference.

^e^Chemsex: sexual intercourse with use of rush (poppers or alkyl nitrites), MDMA (3,4-ethylenedioxymethamphetamine; ecstasy), ice, amphetamines, tramadol, or ketamine in the past six months.

^f^CAI: condomless anal intercourse.

^g^STI: sexually transmitted infection.

^h^HIVST: HIV self-testing.
